# Shaping free-electron radiation via van der Waals heterostructures

**DOI:** 10.1038/s41377-023-01221-3

**Published:** 2023-07-28

**Authors:** Xiao Lin, Hongsheng Chen

**Affiliations:** 1 Interdisciplinary Center for Quantum Information, State Key Laboratory of Extreme Photonics and Instrumentation, ZJU-Hangzhou Global Scientific and Technological Innovation Center, College of Information Science & Electronic Engineering, Zhejiang University, Hangzhou, 310027 China; 2 International Joint Innovation Center, the Electromagnetics Academy at Zhejiang University, Zhejiang University, Haining, 314400 China; 3 Key Laboratory of Advanced Micro/Nano Electronic Devices & Smart Systems of Zhejiang, Jinhua Institute of Zhejiang University, Zhejiang University, Jinhua, 321099 China; 4 Shaoxing Institute of Zhejiang University, Zhejiang University, Shaoxing, 312000 China

**Keywords:** X-rays, Nanophotonics and plasmonics

## Abstract

The van der Waals heterostructures with aperiodic stackings have been exploited to shape the spatiotemporal wavefront of free-electron X-ray radiation.

Free-electron radiation arises from the interaction between fast-moving electrons and matters. It is capable to create the light emission at arbitrary frequency, ranging from microwave, terahertz, infrared, visible, ultraviolet, to X-ray regimes. Due to this unique capability, free-electron radiation has enabled many practical applications, including high-energy particle detectors, particle accelerator, free-electron lasers, electron microscopy, security scanning, bio-medical imaging, to photodynamic therapy. Because of the fundamental significance of free-electron radiation, its continuing exploration and applications actually have gave rise to at least six Nobel prizes in physics, such as those in 1958, 1959, 1988, 1995, 2002, and 2015.

Due to the complexity of light-particle-matter interactions, there are still some long-standing challenges of free-electron radiation that are highly sought after, despite the long research history of free-electron radiation. One challenge is how to flexibly shape the spatiotemporal wavefront of the emitted light during particle-matter interactions, especially at the X-ray regime. Actually, since the refractive index of most optical material is very close to unity at high frequencies, the necessary X-ray optical components (e.g. mirrors and lenses) that can freely mold the flow of light are extremely lacking up to date.

To mitigate this issue, an international team led by Prof. Ido Kaminer from Technion, Israel Institute of Technology, recently proposed a novel paradigm to simultaneously shape the emission and propagation of X-rays by exploring the interaction between free electrons and van der Waals heterostructures with aperiodic (e.g. chirped) interlayer spacings^1^. Despite that the required accuracy of interlayer spacings is in the order of angstrom, these van der Waals heterostructures are in principle realizable in experiments, when considering the recent advances of synthesis and nano-fabrication of atomically-thin materials (e.g. graphene, twisted bilayer graphene, molybdenum disulfide). Essentially, these judiciously-customized interlayer spacings would introduce a desired curved wavefront into the generation process of free-electron X-ray radiation, or the so-called parametric X-ray radiation. This way, the emitted X-rays themselves are capable to be focused, without the aid of additional X-ray optical components. By following this paradigm, they showed that the usage of geometric configuration in the manipulation of parametric X-ray radiation could create X-ray beams with nanoscale focal spot sizes and micrometer-scale focal lengths. Therefore, this proposed paradigm shows an enticing potential to bypass the efficiency limits imposed by current X-ray optical components and opens new possibilities for more applications based on X-rays.

Looking forward, due to the abundance of 2D materials, there are numerous ways to construct van der Waals heterostructures, not only in the vertical direction, but also in the lateral direction. As ignited by the rapid progresses of 2D materials, there are recently renewed interests in the study of free-electron radiation from 2D materials and their heterostructures^2–8^. Rich physics can be expected in this emerging realm, as exemplified by recent works from Kaminer’s group^1,9,10^, and they might open new avenues towards more flexible control of free-electron radiation as schematically shown in Fig. 1, including its frequency, polarization, intensity, directionality, and angular momentum, in addition to its wavefront. However, the study of this emerging realm is still in its infancy. The continuing exploration of free-electron interactions with 2D materials and their heterostructures may further trigger many open questions, concerning, for example, the experimental observation of focused free-electron X-ray radiation, the possibility to further enhance radiation intensity of many other types of free-electron radiation (e.g. Cherenkov radiation, transition radiation, Smith-Purcell radiation), the miniaturization of free-electron based large scientific apparatus (e.g. free-electron lasers, Cherenkov detectors, transition radiation detectors, particle accelerators), and the design of on-chip free-electron light sources with high directivity and high intensity.

**Fig. 1:**
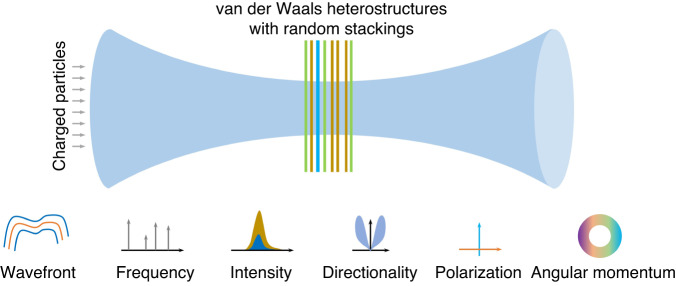
Schematic of controlling of free-electron radiation via van der Waals heterostructures
